# Kissing Lesion of Mitral Valve - A Case Report

**DOI:** 10.21470/1678-9741-2019-0168

**Published:** 2021

**Authors:** Javid Raja, Rupesh Kumar, Krishna Prasad Gourav

**Affiliations:** 1 Department of Cardiothoracic and Vascular Surgery, Postgraduate Institute of Medical Education & Research – PGIMER, Chandigarh, India.; 2 Department of Anesthesia and Intensive Care, Postgraduate Institute of Medical Education & Research – PGIMER, Chandigarh, India.

**Keywords:** Aortic Valve Insufficiency, Endocarditis, Bacterial, Mitral Valve, Prolapse

## Abstract

Aortic valve endocarditis can lead to secondary involvement of aorto-mitral curtain and the adjacent anterior mitral leaflet (AML). The secondary damage to AML is often caused by the infected jet of aortic regurgitation hitting the ventricular surface of the mitral leaflet, or by the pronounced bacterial vegetation that prolapses from the aortic valve into the left ventricular outflow tract. This is called ‘kissing lesion’. We describe a patient with infective endocarditis of the aortic valve causing perforation of both noncoronary cusp of aortic valve and the AML, which is rare.

**Table:** 

Abbreviations, acronyms & symbols	
AML	= Anterior mitral leaflet
AO	= Aorta
AR	= Aortic regurgitation
LA	= Left atrium
LV	= Left ventricle
MR	= Mitral regurgitation
NYHA	= New York Heart Association
RCC	= Right coronary cusp
RV	= Right ventricle
SJM	= St. Jude Medical

## INTRODUCTION

Infective endocarditis of the aortic valve can lead to secondary involvement of aorto-mitral intervalvular fibrosis and anterior mitral leaflet (AML). Aortic lesions usually extend along the continuity of the mitral-aortic structures. The so-called ‘kissing lesion’ is observed in 10–15% of patients with infective endocarditis of the aortic valve^[1]^. In this rare case report, we describe a patient with infective endocarditis of the aortic valve causing perforation of both noncoronary cusp of aortic valve and the AML.

## CASE REPORT

**Fig. 1:**
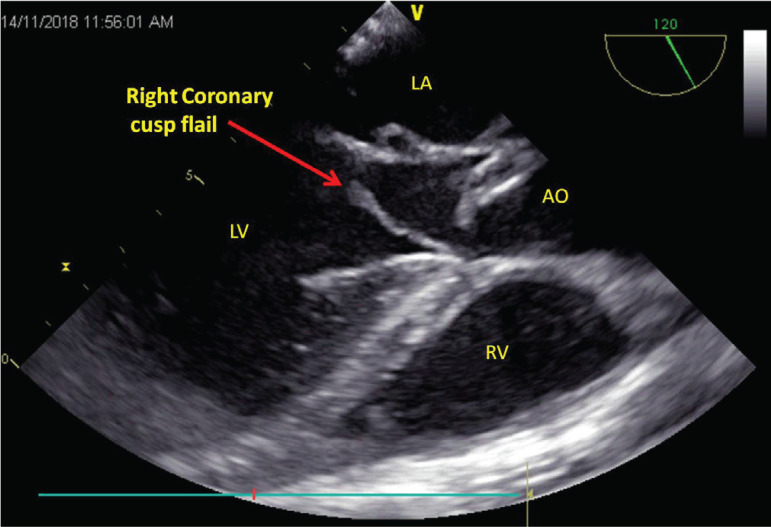
Transesophageal echocardiography showing right coronary cusp flail causing perforation of anterior mitral leaflet. AO=aorta; LA=left atrium; LV=left ventricle; RV=right ventricle

**Fig. 2:**
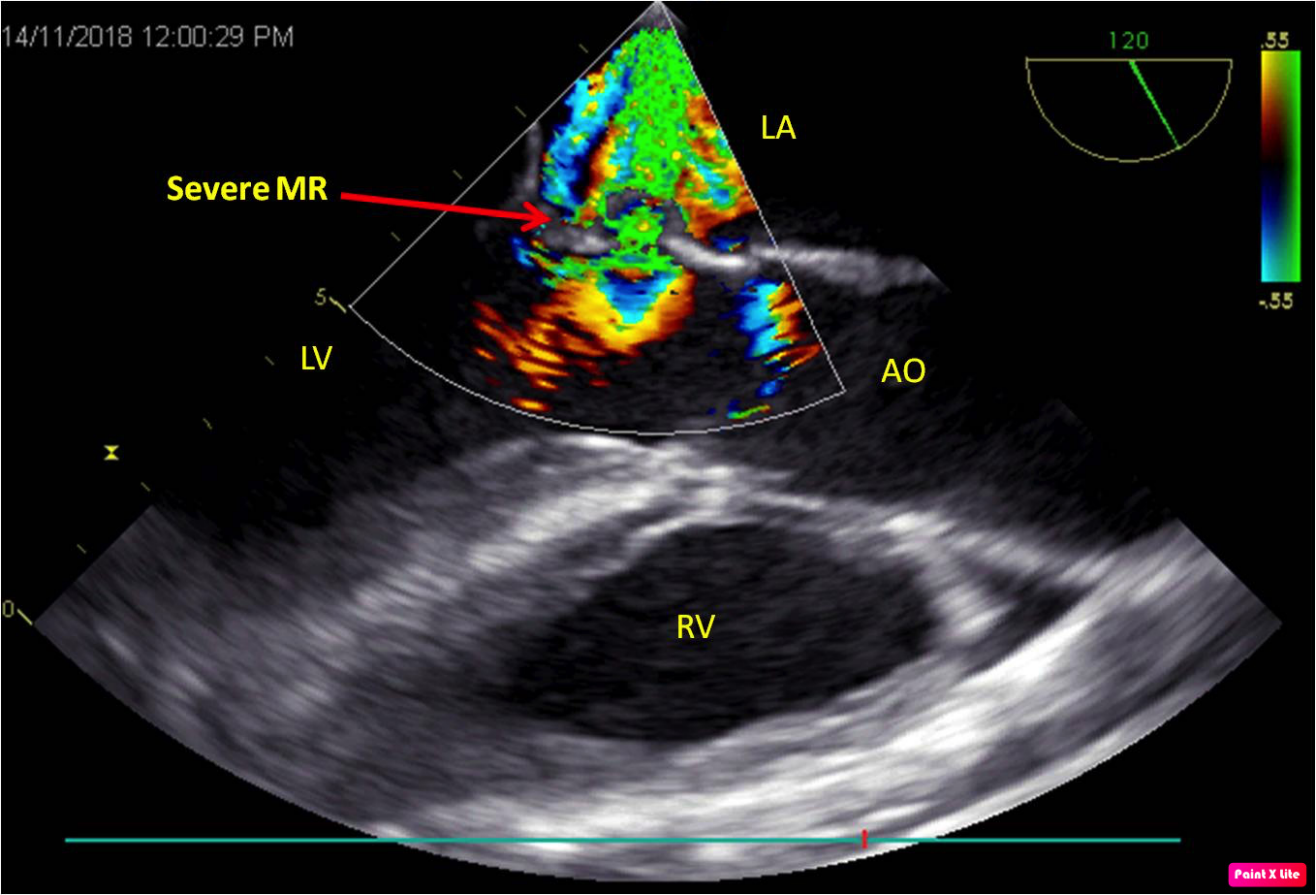
Transesophageal echocardiography showing perforation of anterior mitral leaflet with severe mitral regurgitation (MR). AO=aorta; LA=left atrium; LV=left ventricle; RV=right ventricle

**Fig. 3:**
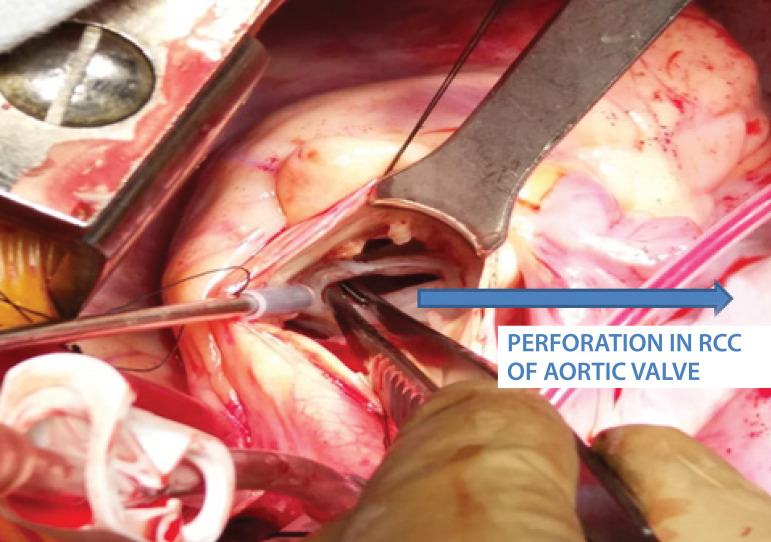
Intraoperative picture showing perforation in the right coronary cusp (RCC) of the aortic valve.

A 32-year-old male patient presented with symptom of breathlessness of New York Heart Association (NYHA) class III for the past one week. He provided a history of fever on and off for the past two months, for which he visited a local hospital and took medications for two weeks and it stopped. There was no history of rashes, chest pain, and syncope. Also, there was no history of drug abuse. On examination, the patient was conscious, oriented, and afebrile, his heart rate was 96/min, a regular rhythm, his blood pressure was 142/52 mmHg, his respiratory rate was 24/min, and there were no other peripheral stigmata of infective endocarditis. Cardiac auscultation revealed a grade 3/6 diastolic murmur in the aortic area and 3/6 pansystolic murmur in the mitral area. Transthoracic echocardiography showed severe aortic regurgitation (AR), severe mitral regurgitation (MR), normal biventricular function, left ventricular end-diastolic diameter of 57 cm, left ventricular end-systolic diameter of 37 cm, effective regurgitant orifice area of 40 mm^2^, and left ventricular volume of 97 ml/m^2^. After performing basic blood investigations, the patient was taken up for double valve replacement. After induction, transesophageal echocardiography showed perforations in noncoronary cusp of aortic valve and AML, causing severe AR and severe MR, respectively. Aortic leaflets showed rupture at the base, with diastolic flail in the left ventricular outflow tract (Figure 1) and kissing of ventricular surface of AML. Figure 2 shows perforation in AML with severe MR. The same findings were confirmed intraoperatively (Figure 3) and the patient successfully underwent aortic valve replacement with 21 mm St. Jude Medical (SJM) mechanical valve and mitral valve replacement with 29 mm SJM mechanical valve. The patient was extubated within six hours and discharged on the 7^th^ day, and he currently is on regular follow-up.

## DISCUSSION

Infective endocarditis of the aortic valve may proliferate onto the adjacent structures^[2]^. Lesions of the mitral valve that are secondary to the aortic infective process are rare and they are known as ‘mitral kissing vegetations’. This type of lesion develops on morphologically and functionally normal leaflet, when the aortic valve vegetation or aortic retrograde flow has a direct contact with the ventricular surface of AML^[3]^.

There are various mechanisms that can be postulated for secondary mitral involvement, which include contiguous spread of the infection from the noncoronary aortic cusp to the ventricular aspect of the neighbouring AML, simultaneous infections of both left heart valves, and, lastly, jet perforation, in which an isolated perforation or infection of the anterior mitral valve cusp occurs as a consequence of a diastolic aortic regurgitant flow impinging on the open AML^[4]^.

Studies have shown that patients with aortic valve endocarditis plus mitral kissing vegetation have higher prevalence of embolic events and renal failure than patients with aortic valve endocarditis alone^[5]^. Aortic valve endocarditis with large vegetations should undergo ‘close’ echocardiographic monitoring, so that with recognition of mitral kissing vegetations surgical intervention can be prompt in order to preserve the integrity of the mitral valve^[6]^.

To conclude, in patients with aortic valve endocarditis, delay in diagnosis and surgery can lead to mitral kissing lesion, thereby increasing the morbidity of the patient as proven by our case.

**Table:** 

Author's roles & responsibilities	
JR	Substantial contributions to the conception or design of the work; or the acquisition, analysis, or interpretation of data for the work; final approval of the version to be published
RK	Drafting the work or revising it critically for important intellectual content; final approval of the version to be published
KPG	The acquisition, analysis, or interpretation of data for the work; final approval of the version to be published
